# Identification of Mortality Risks in the Advancement of Old Age: Application of Proportional Hazard Models Based on the Stepwise Variable Selection and the Bayesian Model Averaging Approach

**DOI:** 10.3390/nu13041098

**Published:** 2021-03-27

**Authors:** Ewelina Łukaszyk, Katarzyna Bień-Barkowska, Barbara Bień

**Affiliations:** 1 Department of Geriatrics, Medical University of Bialystok, Fabryczna 27, 15-471 Bialystok, Poland; bien@umb.edu.pl; 2 Geriatric Ward, Hospital of the Ministry of the Interior and Administration in Bialystok, 15-471 Białystok, Poland; 3 Institute of Econometrics, Warsaw School of Economics, Madalińskiego 6/8, 02-513 Warsaw, Poland; katarzyna.bien@sgh.waw.pl

**Keywords:** survival, geriatrics, vitamin D, TUG, lymphocytes, GNRI, Charlson Comorbidity Index, Bayesian model averaging

## Abstract

Identifying factors that affect mortality requires a robust statistical approach. This study’s objective is to assess an optimal set of variables that are independently associated with the mortality risk of 433 older comorbid adults that have been discharged from the geriatric ward. We used both the stepwise backward variable selection and the iterative Bayesian model averaging (BMA) approaches to the Cox proportional hazards models. Potential predictors of the mortality rate were based on a broad range of clinical data; functional and laboratory tests, including geriatric nutritional risk index (GNRI); lymphocyte count; vitamin D, and the age-weighted Charlson comorbidity index. The results of the multivariable analysis identified seven explanatory variables that are independently associated with the length of survival. The mortality rate was higher in males than in females; it increased with the comorbidity level and C-reactive proteins plasma level but was negatively affected by a person’s mobility, GNRI and lymphocyte count, as well as the vitamin D plasma level.

## 1. Introduction

Older adults are often the most complex and complicated medical patients and, therefore, at high risk for morbidity and mortality. Although advanced age remains the most important risk factor for death [1], it should not be forgotten that chronological age usually is not an equivalent of biological age [2]. Moreover, the importance of conventional risk factors, such as obesity [3,4], high cholesterol concentration, and cardiovascular diseases (including hypertension), tends to decrease in the oldest decades of age [5]. Therefore, there is still a need to look for other possible factors that might affect mortality in the oldest populations.

It is well known that malnutrition is associated with morbidity, and mortality; increased frequency of hospital admissions, prolonged hospital stays, and immune dysfunctions [6,7]. Screening tools for malnutrition, such as the Mini Nutritional Assessment (MNA), are routinely used in comprehensive geriatric assessment [8]. A novel and more precise scoring system has been recently proposed: The Geriatric Nutritional Risk Index (GNRI) [9]. It has been validated in institutionalized patients [10], dialysis patients [11], and patients with heart failure [12]. GNRI scoring includes serum albumin; therefore, it may be better correlated with systemic inflammation in the elderly.

It has been shown that malnutrition affects the immune status, which is manifested by a decrease in the total lymphocyte count [13]. Moreover, low lymphocyte count has been considered as an indicator of immunosenescence in geriatric patients and could be associated with increased mortality risk in the elderly population [14,15]. Together, both GNRI and total lymphocyte count might also improve the evaluation of nutritional risk and could predict short-term health complications [16]. 

Vitamin D deficiency is a global health problem, especially in elderly populations with poor nutritional status. The optimal 25-OH-vitamin D level revealed beneficial effects for patients with numerous illnesses; these include diabetes mellitus, cancer, autoimmune diseases, cognitive function, and even COVID-19 [17]. The vitamin D receptors are expressed by various types of immune cells that include lymphocytes [18]. The research has shown that decreased total lymphocyte count was associated with low levels of 25(OH) vitamin D [19]. However, the evidence of a relationship between vitamin D status and mortality is still inconsistent. 

There is some evidence that the Comprehensive Geriatric Assessment (CGA) in older populations predicts an overall survival rate in cancer patients [20,21]. Physical performance measured by standardized tools, such as Timed Up and Go test (TUG) seems to predict adverse cardiovascular outcomes and mortality in the elderly [22]. Multiple comorbid medical conditions are observed much more often in older adults [23]. Comorbidity is associated with significant health complications, including mortality [24], and is most often assessed using the Charlson Comorbidity Index (ChCI) [25].

Attempting to combine easily identified and accessible indicators of health, functional, and nutritional status, this study’s main objective is to identify an optimal set of predictors that are independently associated with the mortality risk of older comorbid adults formerly hospitalized in a geriatric ward just before the COVID-19 pandemic. As the standard stepwise variable selection procedures that chose one subset of predictors are often criticized for neglecting the model uncertainty, and a *p*-value near 0.05 can offer only weak evidence against the null hypothesis of no effect [26], we additionally used the more statistically robust method of Bayesian model averaging (BMA) [27,28,29,30]. The BMA method appropriately averages over all non-negligible probable models and leads to statistically sound inferences about risk factors for mortality rates. 

## 2. Materials and Methods

### 2.1. Study Design

The design of this study was based on survival analysis of 433 patients that were discharged from the hospital between the end of 2016 and the end of 2018 (298 women and 135 men, aged 62–102, with an average age of 82.4, SD 6.5). The retrospective data were collected from the geriatric teaching unit (17 beds) in a medium-sized hospital—drawing on a local population of over 0.3 million. Patients were admitted to the geriatric ward due to multidimensional treatment needs and a recent aggravation of multifaceted health problems. An average patient presented 6.7 (SD of 2.3) chronic conditions out of 21 defined (ischemic heart disease, hypertension, atrial fibrillation, heart failure, cerebrovascular disease, arthritis, Parkinson disease, depression, dementia, delirium, anemia, diabetes, infection, liver disease, chronic kidney disease, ulcer, thyroid disorder, cancer, benign prostatic hyperplasia, connective tissue disease, chronic obstructive pulmonary disease). The mean length of hospital stays was 7.0 days (SD of 4.0). No exclusion criteria were applied to the study. The hospital records on different sociodemographic and health-related characteristics of geriatric patients were combined with the information about the length of survival time for each person after the hospital discharge. The exact dates of deaths were obtained from the Ministry of Digital Affairs. The censoring date for the survival was 3 March, 2020, i.e., just before the COVID-19 pandemic; up to that date, 132 persons (30.5%) died. The time of survival variable ranged from 1 day to 1594 days (4.4 years), with the median survival time equal to 893 days (about 2.5 years). The study was conducted in accordance with the Declaration of Helsinki, and the protocol was approved by the Ethics Committee of Medical University of Bialystok (Project identification code: R-I-002/602/2018).

### 2.2. Potential Predictors of Mortality Rate

Geriatric patients are persons of advanced old age with complex morbidity; they are in need of comprehensive geriatric assessment due to a recent deterioration of their physical and/or psychical health [31]. A highly qualified team including geriatricians, physiotherapists, psychologists, and nurses diagnosed the patients in line with the CGA guidelines [32]. The hospital records were completed based on the thorough interviews with patients, physical and functional assessment, and laboratory findings. 

The potential explanatory variables (predictors) for the mortality risk of hospitalized older adults included sociodemographic and health-related characteristics (Table 1). These variables included basic sociodemographic features, such as age; gender; mode of living (alone or in an institution versus with their family); number of years spent in education; anthropologic measures, such as weight (kg), and height (in meters); as well as the outcomes of CGA procedures routinely performed on the 1st or 2nd day after admission. More specifically, the functional status, being defined as the ability to complete basic activities of daily living (ADL), was evaluated using the Barthel Index [33]. The total score of the basic ADL ranged from a minimum of 0 (complete dependence) to a maximum of 100 (complete independence). Instrumental ADL (I-ADL) were assessed using the Duke OARS (Older Americans Resources and Services) Assessment [34]. Six I-ADL domains were included: Housework (cleaning floors and other domestic tasks), preparing their own meals, everyday shopping, using the telephone, handling their own money, and taking their own medicines. The summary I-ADL score ranged from 0 (lowest function) to 12 (highest function). The risk of bedsores was assessed with the Norton scale [35] (the lesser the score, the lower the risk) and the undernutrition with the MNA short form [36] (the lesser the score, the higher the risk). Emotional status was evaluated using the 15-item Geriatric Depression Scale [37], with a range of 0–5 showing no depression and 6–15 indicating a rising risk of depression. Cognitive status was assessed using the Modified Short Blessed Test [38], with a range of 0–7 indicating normal or mild cognitive impairment and 8–28 indicating a rising risk for dementia. Lastly, the Mini Mental State Examination [39] with a range 0–30 (the lesser the score, the worse the level of cognitive status is).

In order to determine a patient’s mobility, the TUG test was performed [40]. The TUG test measured the time (in seconds) needed to rise from a chair and walk 3 meters, turn around, walk back to the chair, and sit down (the use of an assistive device was allowed—if needed). The speed of the TUG performance (in m/second) was recalculated for all patients. To this end, the distance of 6 m was divided by the time of TUG test performance in seconds. In order to not exclude from the study most disabled bedridden patients (42 cases), those persons were assigned the value of 0 m/second. 

Major biochemical measurements included: Plasma hemoglobin in g/dL, the total lymphocyte count in K/µL, plasma albumin in g/dL, plasma vitamin B12 in pG/mL, plasma 25(OH)vitamin D in ng/mL, blood level of total cholesterol in mg/dL, C-reactive protein level in mg/L, fasting glucose in mg/dL, creatinine level in mg/dL, and the glomerular filtration rate according to the CKD-EPI formula in mL/minute/1.73 m^2^. Hematological measurements were performed using fresh venous blood with EDTA, as well as clotted blood. 

Apart from the laboratory findings, the GNRI score was calculated according to the Lorentz formula [9]. GNRI is a well-recognized and complex measure of the geriatric nutritional status summarizing information on a patient’s height (in cm) and weight (in kg), as well as the albumin level in g/L [41]. 

Due to a significant number of coexisting conditions in one geriatric patient, comorbidity was measured with the age-weighted ChCI [25]. It ranged from a minimum of 0 to a maximum of 31, depending on the age and presence of selected diseases (including inter alia cardiovascular diseases, diabetes mellitus, dementia, pulmonary disease, cancer) with assigned weights.

### 2.3. Statistical Analysis

A Chi-squared or Mann–Whitney test was used to assess whether there was a significant difference between the distributions of potential explanatory variables that described the patients who died and the patients who survived over the course of the follow-up period. 

The univariable Cox proportional hazards models (Cox PH) were estimated to investigate the statistical significance of the association between each of the 24 sociodemographic or health-oriented characteristics of geriatric patients and the mortality rate. Selected continuous variables were log-transformed if such a nonlinear functional form allowed for a better goodness-of-fit—according to the model log-likelihood value. The level of statistical significance was set as *p* < 0.05.

Next, multivariable Cox PH models were estimated. As the initial set of potential predictors for the mortality rate included up to 24 explanatory variables, we applied and compared 2 alternative methods of variable selection; namely, the standard stepwise backward variable selection procedure and the more computation-intensive iterative BMA method [27,28,29,30,42,43,44]. Contrary to the stepwise variable selection methods, the latter approach represents a coherent procedure that improves upon the uncertainty of the single model [27,42]—especially if there are multiple predictor choices for the mortality rate that might potentially include confounding variables [28]. Therefore, the BMA can allow us to assess whether any redundant variable was inappropriately identified as a significant predictor of the mortality rate. Instead of using one Cox PH model, we implemented an approach proposed by Volinsky et al. (1997) and used a set of Cox PH models for making inferences [28]. The BMA mechanism is based on the appropriate weighted averaging over all such candidate models, wherein each of them includes a different set of explanatory variables. The BMA approach is becoming increasingly popular and, for example, was also used for both gene selection and the classification of microarray data [43] or in medical applications based on logistic regression setup [44,45]. Specifically, the BMA results are usually reported using the mean of parameter estimates from models that are characterized by a sufficiently high posterior probability—given observational data. This mean coefficient corresponding to a given explanatory variable captures the direction and strength of the effect that this predictor exerted on the mortality rate. Another important result is the posterior probability that the parameter corresponding to an explanatory variable is nonzero, P(β ≠ 0|D), where D denotes the data. The descriptive interpretation of obtained values can be the following [28]. If P(β ≠ 0|D) < 0.5, *there is evidence against an effect* of a given predictor on the mortality rate of geriatric inpatients; if 0.5 < P(β ≠ 0|D) < 0.75, there is *weak evidence of an effect*; if 0.75 < P(β ≠ 0|D) < 0.95, *there is positive evidence*; if 0.95 < P(β ≠ 0|D) < 0.99, *there is strong evidence*; and if 0.99 < P(β ≠ 0|D), *there is very strong evidence* that a given predictor exerts an effect on the mortality rate of hospitalized comorbid older adults. The final set of best predictors in our study was obtained using the iterative version of BMA [43], in which only these risk factors with the posterior probability greater than 0.3 were retained in the final Cox PH model.

The predictive performance of the identified set of best predictors was tested with 10-fold cross-validation [46]. To this end, the whole sample, which comprised of 433 observations, was randomly split into 10 nearly equal non-overlapping portions of data (one subset containing 46 observations and each of the other subsets containing 43 observations). In each of the 10 iterations of the cross-validation procedure, nine subsets were jointly used as a training set, whereas the remaining smaller amount of data served as a testing set. Once the models were estimated on a training set, their predictive performance was checked on the remaining testing set of observations. This process was repeated 10 times, and in each of these 10 iterations, different training and testing samples were used. 

The predictive performance of the variables, identified as statistically significant using the backward variable selection method, was evaluated based on the *predictive discrimination ability*; this examined how well this set of variables sorted and classified the subjects in the testing block into the risk categories (lower, medium, and higher mortality risk) [28]. To this end, in each of the 10 iterations of the cross-validation procedure, the model was estimated on the training set to obtain the vector of parameter estimates $\hat{\beta }$ and to calculate *the risk scores*, $i.e.,x_{i}^{T}\hat{\beta }$, where $x_{i}^{T}$ denotes the row vector of covariates for the *i*th subject (i.e., a geriatric patient). The lower-mortality-risk group, the medium-mortality-risk group, and the higher-mortality-risk group of patients were identified based on the 0.33-quantile ($z_{1}$) and 0.66-quantile ($z_{2}$) of the risk scores calculated for all the subjects in the training set. Next, the risk scores were recalculated for the patients from the testing set, and each subject was assigned to the particular risk group based on $z_{1}$ and $z_{2}$ values. More specifically, a patient, characterized by a risk score $x_{i}^{T}\hat{\beta }\;<\;z_{1}$ was classified as the one having a lower mortality risk. A patient, for whom $z_{1}\;\leq \;x_{i}^{T}\hat{\beta }\;<\;z_{2}$, was assigned to a group with a medium mortality risk, and a patient for whom $z_{2}\;\leq \;x_{i}^{T}\hat{\beta }$_,_ was classified as one having a higher mortality risk. 

The predictive discrimination ability of variables, retained as significant using the backward variable selection method, was also compared with the predictive discrimination ability of iterative BMA. In short, in every single replication of the cross-validation procedure, BMA requires fitting to the training set *K* candidate models: *M*_1_, *M*_2_, …, *M_K_* to obtain the corresponding parameter estimates: ${\hat{\beta }}_{1},{\hat{\beta }}_{2}$, …, ${\hat{\beta }}_{K}$, and the corresponding risk scores $x_{i}^{T}{\hat{\beta }}_{1}$, $x_{i}^{T}{\hat{\beta }}_{2}$, …, $x_{i}^{T}{\hat{\beta }}_{K}.$ The mean risk score for a geriatric patient was calculated as a weighted average from *K* competing models: $\sum_{k=1}^{K}x_{i}^{T}{\hat{\beta }}_{K}P\left(M_{k}|D\right)$, where $P\left(M_{k}|D\right)$ denotes the posterior probability of the *k*th model. The 0.33-quantile and the 0.66-quantile of the mean risk scores can be used to classify the patients in the testing set to appropriate risk groups. 

Statistical analyses were performed with STATA software version 15.0 (StataCorp LP, College Station, TX, USA) and R software (version 3.6.3, R Foundation for Statistical Computing, Vienna, Austria; http://www.r-project.org (accessed on 15 April 2020)) with the BMA package for the Bayesian model-averaging (authors: Raftery, A., Hoeting, J., Volinsky, C., Painter, I., Yeung, K.Y.; https://cran.r-project.org/web/packages/BMA (accessed on 15 April 2020)) [47].

## 3. Results

**Figure 1:**
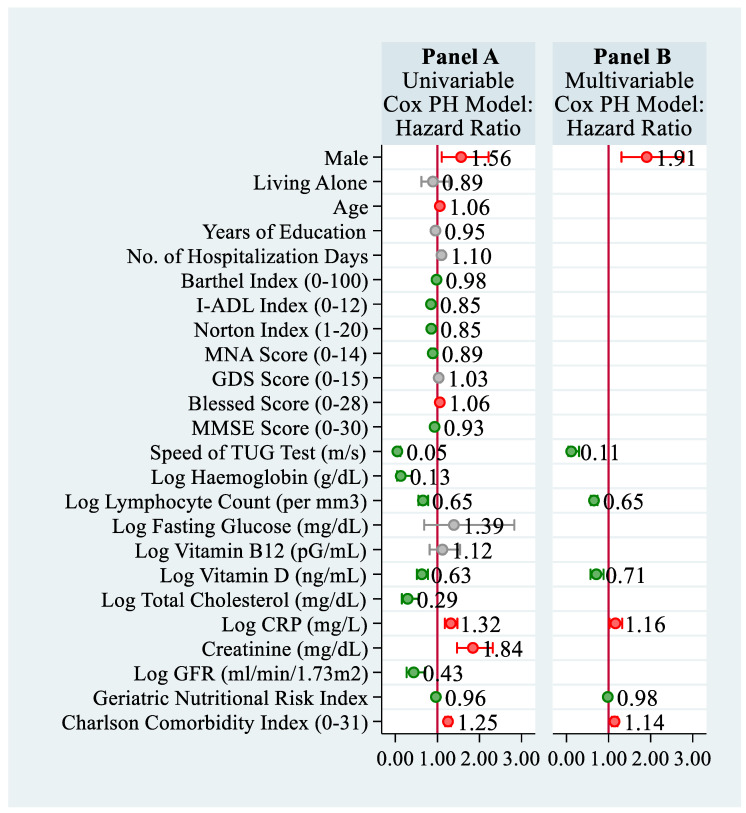
Association between the sociodemographic and health-related characteristics of former geriatric patients and their mortality rate: Results from the univariable Cox PH model (panel **A**) and the multivariable Cox PH model obtained with the stepwise backward variable selection method with the significance level set as *p* < 0.05 (panel **B**).

Estimation results for the univariable Cox PH models are illustrated in Figure 1 (panel A). Accordingly, the mortality risk of former geriatric patients was significantly higher in males than in females (HR = 1.56; *p* = 0.012; 95% CI: [1.10, 2.21]), increased with the number of hospitalization days (HR = 1.1; *p* < 0.001; 95% CI: [1.06, 1.14]), patient’s age (HR = 1.06; *p* < 0.001; 95% CI: [1.03, 1.09]), the Modified Short Blessed Test score (HR = 1.06; *p* < 0.001; 95% CI: [1.04, 1.08]), log CRP level (HR = 1.32; *p* < 0.001; 95% CI: [1.18, 1.48]), plasma creatinine level (HR = 1.84; *p* < 0.001; 95% CI: [1.47, 2.32]), and patient’s comorbidity measured with the age-weighted ChCI (HR = 1.25; *p* < 0.001; 95% CI: [1.18, 1.32]). On the other hand, the scores on the Barthel Index (HR = 0.98; *p* < 0.001; 95% CI: [0.97, 0.98]) or the I-ADL index (HR = 0.85; *p* < 0.001; 95% CI: [0.81, 0.89]) were significantly negatively related to the patient’s mortality rate. Moreover, the mortality risk decreased with a patient’s Norton score (HR = 0.85; *p* < 0.001; 95% CI: [0.82, 0.89]), the MNA (HR = 0.89; *p* < 0.001; 95% CI: [0.84, 0.94]), MMSE score (HR = 0.93; *p* < 0.001; 95% CI: [0.91, 0.95]), the GNRI (HR = 0.96; *p* < 0.001; 95% CI: [0.95, 0.98]), the mobility level measured by the TUG speed (HR = 0.05; *p* < 0.001; 95% CI: [0.02, 0.12]), and selected biochemical measurements: the log hemoglobin level (HR = 0.13; *p* < 0.001; 95% CI: [0.04, 0.40]), log total lymphocyte count (HR = 0.65; *p* < 0.001; 95% CI: [0.57, 0.76]), the log total cholesterol level (HR = 0.29; *p* < 0.001; 95% CI: [0.15, 0.55]), log GFR (HR = 0.43; *p* = 0.001; 95% CI: [0.27, 0.71]), and the log vitamin D level (HR = 0.63; *p* < 0.001; 95% CI: [0.51, 0.78]). All other explanatory variables were not significantly related to the patient’s mortality rate.

**Figure 2:**
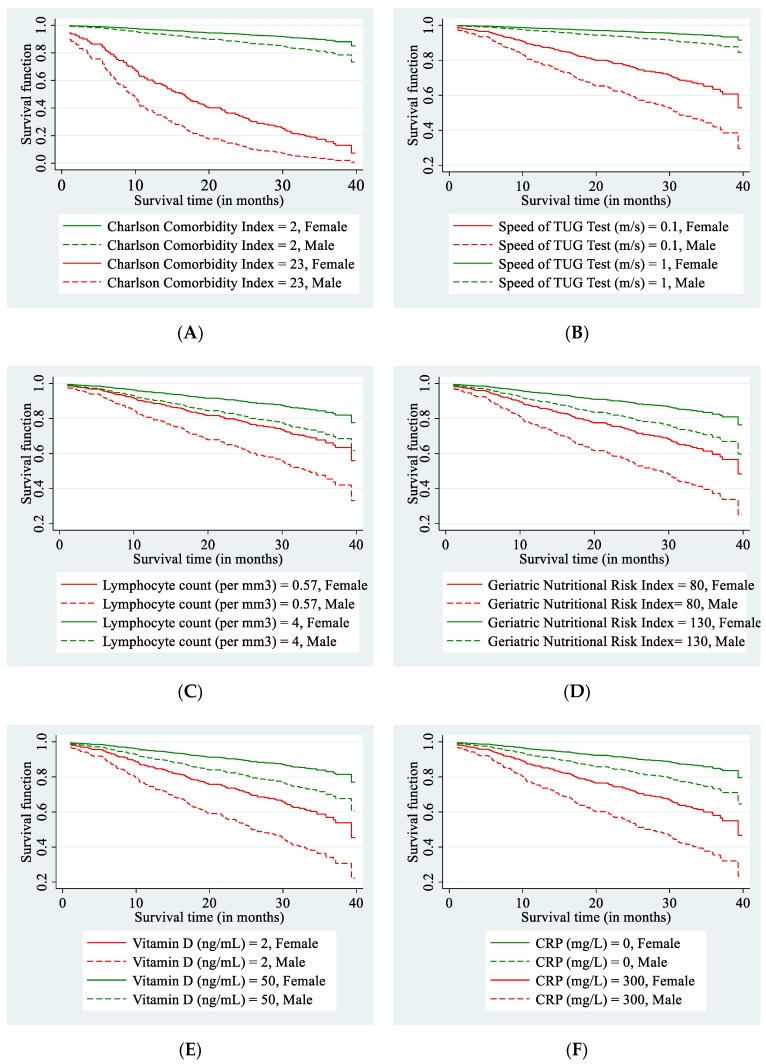
Survival curves for a geriatric patient depending on his or her sex and the selected health-related covariates identified using the stepwise backward variable selection approach (with *p* < 0.05): Age-weighted Charlson Comorbidity Index (panel **A**), the speed of performing the Timed Up and Go (TUG) test (panel **B**), total lymphocyte count (panel **C**), Geriatric Nutritional Risk Index (panel **D**), the vitamin D level (panel **E**), and CRP level (panel **F**). For each panel, the survival curves were derived from the multivariable Cox PH model for given sex and for selected very low or very high values of one explanatory variable, whereas the values of remaining covariates in the model were set at their average level.

The stepwise backward variable selection method that was applied to the multivariable Cox PH model retained seven explanatory variables that were independently and significantly associated with the roughly 2.5-year survival of older adults (Figure 1, panel B). We can see that the mortality rate was significantly higher in males than in females (HR = 1.91; *p* < 0.001; 95% CI: [1.33, 2.76]); it increased with the comorbidity status measured with the age-weighted ChCI (HR = 1.14; *p* < 0.001; 95% CI: [1.07, 1.21]) and, besides this, it also increased with the log C-reactive protein—indicating an inflammatory condition (HR = 1.16; *p* = 0.014; 95% CI: [1.03, 1.30]). The nutritional status of an older adult that was evaluated using GNRI (HR = 0.98; *p* = 0.003; 95% CI: [0.97, 0.99]) and log total lymphocyte count (HR = 0.65; *p* < 0.001; 95% CI: [0.53, 0.80]) also had an independent beneficial effect on survival, as they both were negatively associated with the mortality rate. Additionally, the mortality risk turned out to decrease with the increasing speed of performing the TUG test (HR = 0.11; *p* < 0.001; 95% CI: [0.04, 0.29]) and the rising log vitamin D level (HR = 0.71; *p* = 0.002; 95% CI: [0.58, 0.88]), whereas the critically low vitamin D level was especially dangerous (Figure 2, panel E). Figure 2 illustrates the obtained association between each of these variables and the length of survival for the men and women, separately. The individual survival curves were computed from the estimated multivariable Cox models at the selected value of one predictor, whereas the remaining covariates were set at their average levels.

According to the iterative BMA method, the multivariable Cox PH model that achieved the highest posterior probability, given the observational data, was the same as the Cox PH model obtained with the backward stepwise variable selection method. The application of the iterative BMA also resulted in the same combination of mortality predictors (Table 2). Accordingly, there was *very strong evidence* that being a male was associated with a higher mortality, i.e., P(β ≠ 0|D) = 1. There was also *very strong evidence* that scale of comorbidity measured with the age-weighted ChCI increased mortality risk, i.e., P(β ≠ 0|D) = 1. The BMA results also clearly confirmed that the higher the (log) speed of performing the TUG test, the lesser the mortality risk was, i.e., *there is very strong evidence of an effect,* P(β ≠ 0|D) = 1. Controlling for the impact of other covariates, the nutritional status of an older comorbid adult, which could be captured by the (log) total lymphocyte count and the GNRI, turned out to have a beneficial effect on survival, i.e., there was *very strong evidence of an effect* for (log) total lymphocyte count, P(β ≠ 0|D) = 1, and there was *positive evidence of an impact* for the GNRI, P(β ≠ 0|D) = 0.923, correspondingly. Having adjusted for other factors, there was also *positive evidence* that a rising level of (log) vitamin D had an additional independent beneficial impact on alleviating mortality risk, i.e., P(β ≠ 0|D) = 0.916). *Only weak evidence of an effect* had been obtained with respect to an impact of a rising (log) CRP level on mortality, i.e., P(β ≠ 0|D) = 0.689.

**Figure 3:**
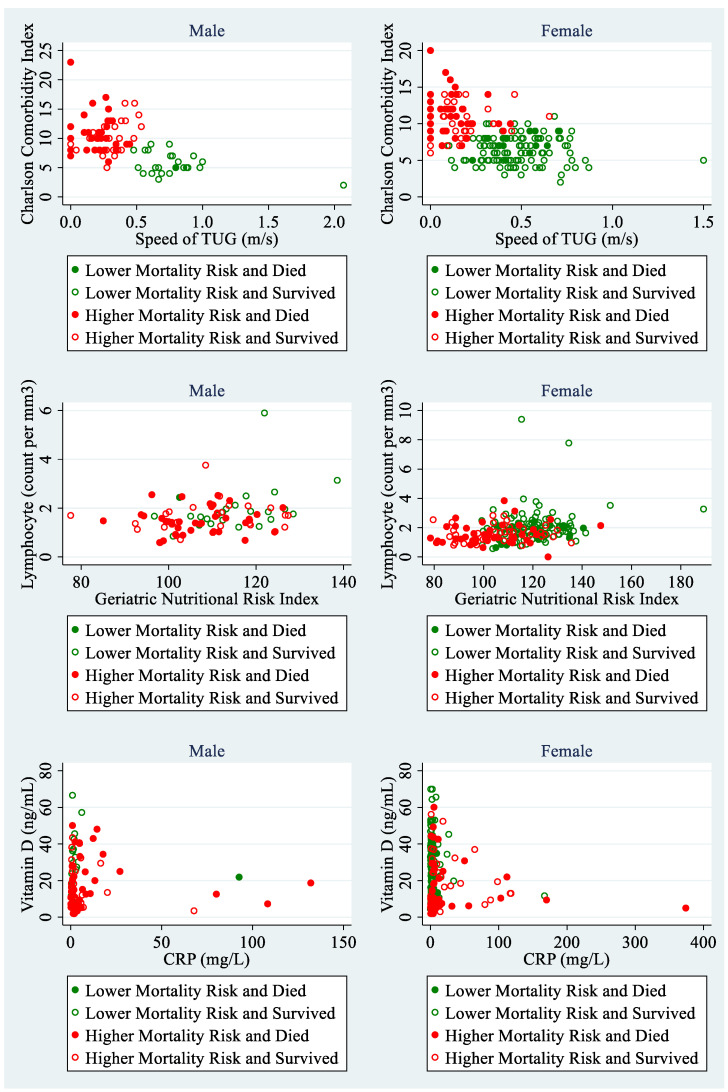
Classification of geriatric patients into the category of lower mortality risk and the category of higher mortality risk based on seven most important predictors identified by both: The stepwise backward selection method and the iterative BMA approach. Results from the 10-fold cross-validation of the multivariable Cox PH model.

**Figure 4:**
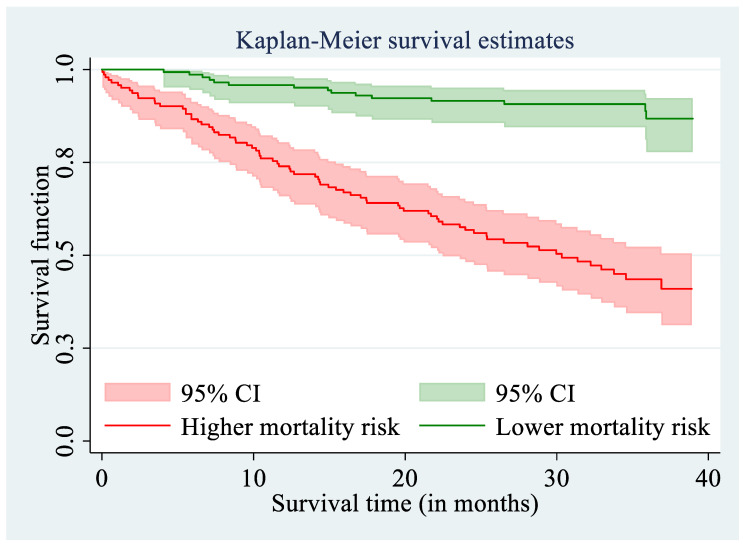
Kaplan–Meier survival curves for patients assigned to a higher and a lower mortality risk category based on a 10-fold cross-validation. Results of the multivariable Cox PH model with the same seven most important risk factors for mortality rate identified by both: The stepwise backward variable selection and the iterative BMA approach.

The predictive performance of the final multivariable Cox PH model (including seven previously identified variables: Being male, age-weighted Charlson Comorbidity Index, speed of TUG test, log total lymphocyte count, Geriatric Nutritional Risk index, log vitamin D, and log CRP) was remarkably good. Out of patients assigned to a lower mortality risk, only 15 (10.6%) patients died, whereas out of patients assigned to a higher mortality risk category, 74 (50%) died. The discrimination accuracy for this set of predictors was only slightly worse than if using the BMA approach that weights the risk scores from competing models. For BMA, out of the 142 patients assigned to a lower risk category, only 15 (10.6%) died; and out of the 148 patients in the higher risk category, 76 died (51.4%). Hence, in two patients, the weighted average of different candidate models improved the predictive accuracy of classification. The predicted classification of former geriatric patients into the lower and higher categories of mortality risk was illustrated in Figure 3, where the individual scatterplots present the impact of two paired continuous or polytomous predictors (out of six identified as most important) for men or women separately. Identification of subjects at higher mortality risk is most visible for patients with low speed of performing the TUG test and high comorbidity level. These less mobile and more comorbid patients were much more often assigned to a higher-mortality-risk group, and indeed, they more frequently died during the 2.5-year period after being discharged from the hospital. The summary of the predictive performance for the Cox PH model with the seven most important covariates is presented in Figure 4. A combination of seven identified predictors for mortality rate successfully discriminates between patients at the lower and higher risk of death over the next 2.5 years.

## 4. Discussion

The extension of life expectancy is driven inter alia by a decline in death rates amongst older people [48]. In recent decades, older people have lived longer and less disabled, despite the higher rate of morbidity controlled by more and more effective treatments [49]. Therefore, we have undertaken an assessment of the factors that determine the respectively longer or shorter survival time in older and comorbid adults.

The presented study identified seven significant factors independently associated with the mortality rate in community-dwelling older adults with high comorbidity. They included: Gender, age-related comorbidity measured with the age-weighted Charlson Comorbidity Index, physical performance that was assessed by the TUG test, immunity state (total lymphocyte count and vitamin D plasma level), protein-energy status assessed by the Geriatric Nutritional Risk Index, and inflammation measured with C-reactive proteins. Beyond gender, the set of contributors covers numerous potentials for survival modification. Although each of the relevant factors interplays with others, they independently affect the organism’s resources. These include: Body structure and its functionality, nutrition as a protein-energetic reserve, and both immunologic and anti-inflammatory capacity [50]. According to the current knowledge about mechanisms of aging [51], they also contribute to the stability of the homeostasis in an aging individual. 

According to the BMA method, only four out of seven factors, namely male gender, age-weighted comorbidity, speed of the TUG performance, and a total lymphocyte count achieved the highest probability unambiguously in explaining the risk of mortality over 2.5 years in multi-morbid older adults.

Gender and age are widely known unmodifiable contributors to the mortality rate. Women outlive men at all ages; however, they show much lower health indices than men of the same age in all industrialized countries, which creates the so-called “male-female-health-survival paradox” [52]. It has also been confirmed in our analysis (Table 2).

Other strong predictors of mortality, such as comorbidity [53] and reduced mobility [54], have long been known and evident. Older adults present an enormous amount of phenotypic diversity [55], huge complexity, and heterogeneity of clinical pictures [56]. Moreover, the geriatric patients are, to some extent, a selected population due to attrition rates resulting from the age-independent mortality before reaching old age [57]. Our previous results [58], as well as those presented in this study, confirm this pragmatic statement about the shorter survival rate of comorbid older adults. The age-weighted Charlson Comorbidity index combines typical age-related morbidity that appears with the advancement of chronological age [25]. This single indicator covers a wide range of age-related morbidities; these include cardiovascular and cerebrovascular chronic conditions, dementias of any origin, diabetes mellitus at different stages, chronic kidney disease, chronic obstructive pulmonary disease, cancer, and many others. Can comorbidity be potentially considered a modifiable target for prolonging patient’s survival? According to a well-known maxim ‘*what has happened cannot be undone*’, the answer is pessimistic. However, from a different perspective, many of these age-related disorders could be potentially preventable. One can find such data in relation to the main killers of older adults, namely cardiovascular diseases [59], as well as dementia [60]. If their risk factors were identified early in life and eliminated, then the survival time should increase, and the overall quality of life in older age should improve.

The speed of the TUG test performance used in this study was found to be as important as the aforementioned factors for explaining survival in the oldest and comorbid populations. The speed of completing any task appears to be a universal indicator of biological aging—regardless of the cause. It combines the outcomes of normal aging like age-related sarcopenia, osteopenia, and immunosenescence, as well as the accumulation in life course pathology like multimorbidities and the deficits of nutritional status. It is worth emphasizing that the positive relationship between walking speed and vitamin D levels was found in the meta-analysis of older adults [61].

Total lymphocyte count is an easily obtained nutrition maker [62] and is, at the same time, the indicator of the efficiency of the immune system—even in younger adults [63]. Lymphopenia was associated with a shorter survival that is independent of traditional risk factors in the 12-year follow-up observational study covering over 31,000 participants [64]. In our study, low total lymphocyte count was also strongly associated with a higher risk of mortality that is independent of other factors, which is in line with previous studies [14,15]. 

Undernutrition is one of the potentially modifiable factors directly associated with life expectancy [65]. It is often an under-recognized condition in hospitalized older adults [66]. GNRI combines albumin’s plasma level (protein-energy status) with body weight and, for this reason, was developed for the geriatric population to indicate nutrition-related complications [9]. Moreover, GNRI was revealed as a good predictor of muscle strength in institutionalized older patients [67], as well as a specific and sensitive tool used in detecting frailty and sarcopenia [68]. According to a recent study by Yuan Y. et al. [69], the low GNRI not only prognoses disease-related complications and mortality but also shows worse exercise tolerance in patients with COPD [70]. The iterative BMA method used in this study showed that there is positive evidence of an independent GNRI effect on the mortality risk in old and comorbid adults. It would be worth considering to evaluate the utility of using GNRI and total lymphocyte count as complex and predictive measures for assessing the risks of health complications [16]. 

Nowadays, hypovitaminosis D is a global health problem [71]. In many observational studies, 25(OH)vitamin D deficiency was correlated with diabetes, cancer, obesity, hypertension, cardiovascular diseases, and cognitive dysfunction [72]. Vitamin D also plays an important role in immune system activity [73]. Mao X. et al. [19] have also elucidated the correlation between lymphocyte subsets and 25 (OH)vitamin D in older adults with age-related diseases. In our research, an insufficient (i.e., very low) concentration of 25(OH)vitamin D turned out to be an independent explanatory variable for the increased mortality in elderly comorbid adults. However, despite the great interest and numerous studies on the effect of vitamin D on survival, the results are still inconclusive [74,75].

Low-grade inflammation, defined as elevated CRP concentration, appears to be associated with an increased risk of mortality in older adults [76]. Moreover, higher inflammatory parameters were observed in older people with frailty [77]. These findings stay in line with our results, although according to the iterative BMA method, we have obtained only weak evidence of the association between the CRP and the mortality risk. 

It is remarkable that the same set of factors, including male gender and comorbidity, is associated with the risk of severe outbreak or death in patients with the COVID-19 disease [78]. There is also some evidence that adequate vitamin D concentration has shown beneficial effects in the SARS-Cov-2 infection [79,80]. Therefore, medical trials are currently being conducted in older adults diagnosed with COVID-19 [81]. GNRI may also be used as a predictive tool for high-risk elderly patients with COVID-19, as well as other nutritional scales [82]. Moreover, in older adults infected with SARS-Cov2, lymphopenia occurs more frequently—especially in severe cases [83,84]. 

Hence, the question arises: Can the accumulation of factors identified in our study be considered a kind of universal sign of multi-organ failure and impending death? Although almost all identified predictors of the over 2-year survival are independently associated with the mortality rate, they interact with each other. Comorbidity usually deteriorates physical activities, nutrition, vitamin D status, immunity, and vice versa. This might suggest that supplementing older people with a diet containing protein-energy ingredients and vitamin D would have a positive effect on immunity and physical activity, and, as a result, would equate to longer survival rates in older adults. 

The limitations of this analysis lie in the relatively small sample and the short time of observation. It should also be added that many other measures and indices not included in this study might determine mortality. Moreover, because the study design is not experimental but observational, the dependencies investigated in this research should be confirmed in randomized controlled trials based on the optimization of the nutritional and functional status of geriatric patients. However, the application of the iterative BMA approach, which accounts for the model uncertainty neglected by the solely stepwise variable selection methods, reinforces the reliability of answers posed to the question under study. 

## 5. Conclusions

This study identifies the best combination of risk factors for all-cause mortality in older comorbid adults formerly hospitalized in the geriatric ward due to recent deterioration of their physical and/or psychical health. The mortality rate turned out to be independently associated with gender, age, comorbidity, mobility, as well as nutritional and immunologic body’s reserves. 25(OH)vitamin D plasma level is also shown to play a protective role for the oldest and comorbid people. Therefore, the recommendations for older adults should focus on optimal nutrition, maintenance or enrichment immunity with better vitamin D status, physical activity, and the prevention of chronic (specifically inflammatory) diseases [85]. However, further research into these factors should still be actively pursued. There is a need for further prospective studies based on the population of the oldest comorbid adults, thus far usually excluded from clinical randomized control trials.

## Figures and Tables

**Table 1:** Descriptive statistics of geriatric patients.

	All Patients *N* = 433	Died *N* = 132	Survived *N* = 301	*p*-Value ^a^
Male, n (%)	135 (31.18)	53 (40.15)	82 (27.24)	0.007
Living Alone, n (%)	148 (34.18)	42 (31.82)	106 (35.22)	0.492
Age, mean (SD ^b^)	82.38 (6.54)	84.17 (6.31)	81.59 (6.49)	<0.001
Years of education, mean (SD)	9.28 (4.21)	8.55 (4.13)	9.60 (4.21)	0.009
No. of hospitalization days (SD)	7.02 (4.01)	7.95 (5.46)	6.61 (3.1)	0.0263
Barthel Index (0–100), mean (SD)	79.88 (23.88)	68.33 (28.50)	84.95 (19.54)	<0.001
I-ADL Index (0–12), mean (SD)	6.53 (3.88)	4.65 (3.82)	7.36 (3.61)	<0.001
Norton Index (1–20), mean (SD)	16.79 (2.86)	15.47 (3.19)	17.36 (2.49)	<0.001
MNA Score (0–14), mean (SD)	10.95 (2.76)	10.21 (2.88)	11.27 (2.64)	<0.001
Geriatric Depression Scale Score (0–15), mean (SD)	6.02 (3.84)	6.33 (4.01)	5.88 (3.76)	0.330
Blessed Score (0–28), mean (SD)	11.03 (9.13)	14.70 (9.81)	9.43 (8.34)	<0.001
MMSE Score (0–30), mean (SD)	20.70 (7.33)	17.57 (8.44)	22.07 (6.33)	<0.001
Speed of TUG test (m/s), mean (SD)	0.34 (0.24)	0.24 (0.19)	0.39 (0.24)	<0.001
Hemoglobin (g/dL), mean (SD)	12.61 (1.66)	12.24 (1.78)	12.77 (1.59)	0.006
Total Lymphocyte Count (K/µL), mean (SD)	1.75 (0.80)	1.56 (0.59)	1.83 (0.85)	<0.001
Fasting Glucose (mg/dL), mean (SD)	107.62 (30.33)	108.78 (26.42)	107.12 (31.93)	0.526
Vitamin B12 (pG/mL), mean (SD)	415.58 (298.61)	427.04 (315.66)	410.55 (291.22)	0.642
Vitamin D (ng/mL), mean (SD)	22.94 (15.34)	18.66 (13.83)	24.82 (15.61)	<0.001
Total Cholesterol (mg/dL), mean (SD)	174.78 (45.80)	164.23 (46.29)	179.40 (44.88)	<0.001
CRP (mg/L), mean (SD)	9.36 (28.08)	15.71 (42.89)	6.57 (17.50)	<0.001
Creatinine (mg/dL), mean (SD)	0.92 (0.45)	1.02 (0.69)	0.88 (0.28)	0.031
GFR (ml/min/1.73 m^2^), mean (SD)	67.38 (18.75)	64.23 (19.88)	68.76 (18.09)	<0.001
Geriatric Nutritional Risk Index, mean (SD)	113.56 (13.28)	108.75 (13.15)	115.67 (12.79)	<0.001
Age-weighted Charlson Comorbidity Index (1–31), mean (SD)	8.30 (2.92)	9.65 (3.18)	7.71 (2.59)	<0.001

^a^ Chi-square test or Mann–Whitney test for no difference between the two distributions (for died and survived), as appropriate; ^b^ SD denotes standard deviation.

**Table 2:** Results from the iterated Bayesian model averaging of the multivariable Cox PH regressions. Posterior parameter estimates (averages), their standard deviations, and probabilities that coefficients are nonzero for the seven identified variables affecting mortality rate.

Predictors	Average Coefficient	Standard Deviation of Coefficients	P(β ≠ 0 | D)
Male	0.632	0.189	1.000
Age-weighted Charlson Comorbidity Index	0.139	0.032	1.000
Speed of TUG Test	−2.255	0.502	1.000
Log Total Lymphocyte Count	−0.423	0.105	1.000
Geriatric Nutritional Risk Index	−0.020	0.009	0.923
Log Vitamin D	−0.309	0.140	0.916
Log CRP	0.106	0.088	0.689

## Data Availability

The data presented in this study are not publicly available due to thefact of confidentiality reasons. These data are available on request from the Prof. Barbara Bien (bien@umb.edu.pl).
